# Prevalence, risk and protective indicators of common mental disorders among young people living with HIV compared to their uninfected peers from the Kenyan coast: a cross-sectional study

**DOI:** 10.1186/s12888-021-03079-4

**Published:** 2021-02-10

**Authors:** Moses K. Nyongesa, Paul Mwangi, Michael Kinuthia, Amin S. Hassan, Hans M. Koot, Pim Cuijpers, Charles R. J. C. Newton, Amina Abubakar

**Affiliations:** 1 KEMRI-Wellcome Trust Research Programme, Centre for Geographic Medicine Research (Coast), KEMRI, Box 230, Kilifi, Kenya; 2 Department of Clinical, Neuro- and Developmental Psychology, Amsterdam Public Health Research Institute, Vrije Universiteit Amsterdam, Amsterdam, Netherlands; 3 Department of Public Health, Pwani University, Kilifi, Kenya; 4 Department of Psychiatry, University of Oxford, Oxford, UK; 5 Institute for Human Development, Aga Khan University, Nairobi, Kenya

**Keywords:** Common mental disorders, HIV infections, Young people, Prevalence, Correlates

## Abstract

**Background:**

In sub-Saharan Africa, common mental disorders (CMDs) like depression and anxiety are under-investigated amongst young people living with HIV (YLWH). To address the gap, in Kenya we: a) determined the prevalence of CMDs among YLWH compared to their uninfected peers; b) investigated HIV status as an independent predictor of CMDs in young people; c) investigated CMDs risk and protective indicators with more focus on YLWH.

**Methods:**

Between November 2018 and September 2019, 819 young people aged 18–24 years (407 HIV-infected) were recruited from two Counties on the Kenyan coast. Locally adapted pre-existing mental health measures, Patient Health Questionnaire (9-item) and Generalized Anxiety Disorder scale (7-item), were administered among other questionnaires via audio computer-assisted self-interview. Logistic regression was used to determine the correlates of CMDs.

**Results:**

Prevalence of CMDs was significantly elevated among YLWH compared to their uninfected peers i.e. *29%* vs. *12%; p < 0.001* for depressive symptoms, *19%* vs. *8%; p < 0.001* for anxiety symptoms, and *16%* vs. *5%; p < 0.001* for comorbid depressive and anxiety symptoms. HIV status independently predicted depressive symptoms and its co-occurrence with anxiety symptoms. Among YLWH, negative life events, higher perceived HIV-related stigma and low adherence to antiretroviral therapy were the risk indicators for elevated CMDs. Among HIV-uninfected youths, death of both parents was a risk indicator for elevated depressive symptoms. Protective indicators against CMDs among youths with and without HIV included higher social support and health-related quality of life.

**Conclusion:**

At the Kenyan coast, YLWH have significantly higher burden of CMDs compared to their uninfected peers. Being HIV-positive as a youth in this setting is predictive of more depressive symptoms and its comorbidity with anxiety symptoms. YLWH at high risk of CMDs in coastal Kenya can benefit from early detection, referral and treatment if routine screening for CMDs is integrated in their care package. The mental wellbeing of bereaving HIV-unaffected youths could be improved through continued support to help them come to terms with their loss. At the community level, programmes strengthening the social capital or improving the overall quality of life of youths with or without HIV may be beneficial to their mental health.

## Background

Currently, young people below the age of 25 years constitute 42% of the world’s population, with the vast majority of them residing in resource-limited settings [1]. These young people are disproportionately affected by the HIV/AIDS epidemic. Globally, an estimated 3.9 million young people aged 15–24 years are living with HIV [2], more than three-quarters of them residing in sub-Saharan Africa [3]. Between 2005 and 2016, the number of young people living with HIV (YLWH) rose by 30% globally [4]. This rising trend could result from the improved survival of YLWH on combined anti-retroviral therapy (cART), where perinatally HIV-infected children are reaching adolescence and young adulthood in large numbers [5, 6]. New HIV infections may also be contributing to the rising number of YLWH. It is reported that nearly half of new HIV infections are occurring in young people [7]. In Kenya, for instance, young people (15–24 years old) were estimated to contribute over half of new adult HIV infections in the year 2015 [8].

With increased survival, many more YLWH will develop mental health problems [5]. This may stem from normative challenges coping with developmental changes occurring in adolescence across the physical, cognitive, emotional and social domains; as well as challenges associated with transitioning from adolescence to adulthood and school- or work-related stressors. However, for YLWH, normative challenges may be complicated by HIV-specific challenges such as adjustment difficulty to living with a chronic illness as a young person, the challenge of adhering to lifetime cART medication and coping with prospective HIV-related stigma, which may lead to additional psychological distress [9–11].

In the last decade, comorbidity of HIV/AIDS and common mental disorders (CMDs), mainly depression and anxiety, has been well described among adults living with HIV, including those from sub-Saharan Africa where HIV burden is high [12, 13]. In contrast, among YLWH, especially those from sub-Saharan Africa, there is limited understanding of this comorbidity [14, 15]. A systematic review of the literature, including 38 studies mostly from Europe and North America, reports a prevalence of CMDs of over 30% among YLWH [5]. Emerging studies from sub-Saharan Africa also report a high prevalence of CMDs among YLWH, ranging from 18 to 53% for depressive symptoms [16–18] and from 5 to 25% for anxiety disorders [18, 19].

Correlates of CMDs among YLWH are hard to delineate because few studies on this topic have been conducted thus far and the findings are inconsistent. Generally, the investigated correlates include both risk and protective indicators and can be categorized as demographic, psychosocial, or HIV-related clinical factors. According to the review of studies mostly from Europe and North America [5], older age and being female were the frequently reported demographic risk indicators for elevated CMDs among YLWH. This finding is also emerging from studies conducted in sub-Saharan Africa [14, 16, 20]. Even so, the male sex has been reported as a predictor of higher depressive scores in YLWH by a study conducted in England [21]. HIV-related stigma among YLWH appears an important psychosocial risk indicator for elevated CMDs from recent African-based reports [14, 20, 22], a finding also observed elsewhere [23]. Other identified psychosocial risk indicators for elevated CMDs among YLWH, but with little or no consensus from past studies, include an experience of negative or stressful life events [24], poor adherence to cART [5, 20], HIV clinic inaccessibility [15] and disclosure of HIV status [25]. Similarly, several HIV-related clinical factors have been investigated as risk indicators for elevated CMDs among YLWH but with little or no consensus. These include low cluster of differentiation-4 cell count [23], presence of HIV opportunistic infections [20], cART regimen particularly efavirenz use [26], duration on cART [9] and its side effects [22]. The role of HIV status (being positive or not) in increasing the risk for CMDS among YLWH also remains unclear as studies report mixed findings [21, 27, 28].

The literature on protective indicators against CMDs in YLWH is particularly scanty. The few published studies report individual-level factors such as better overall health [22], nutritional status [26], and satisfaction with physical appearance [25] as significantly lowering the risk of CMDs among YLWH. Psychosocial factors such as higher social support [17, 22], self-efficacy [22] and positive parenting [22] have been identified as protective against CMDs among YLWH in Africa, but by a single study or studies from the same country (South Africa). We are not aware of published research, specifically from other African countries, corroborating any of these findings. It remains unclear whether better quality of life may be protective against CMDs comorbid with HIV in young people, but a significant inverse association has been observed between quality of life and depressive symptoms among adults living with HIV in the African setting and beyond [29, 30]. Of the HIV-related factors, the absence of immunosuppression among YLWH [25] has been reported as a protective indicator against CMDs.

In sub-Saharan Africa, investigations of the burden and factors associated with CMDs among YLWH are generally limited compared to similar research among adults with HIV. Of the existing reports from this setting, very few have simultaneously investigated the burden of multiple CMDs in YLWH [24] or enrolled an appropriate control group for comparison purposes [24, 28]. There are inconsistencies too across studies in terms of reported correlates of CMDs among YLWH. More insight on the contextual factors influencing the mental health of YLWH in the African context is important to inform evidence-based interventions. As an addition to the limited and unclear body of knowledge, the present study from the Kenyan coast was designed to: i) determine the prevalence of CMDs, specifically depressive symptoms and generalized anxiety disorder symptoms, among YLWH compared to their uninfected peers; ii) investigate HIV status as an independent predictor of CMDs in a sample of young people; iii) investigate CMDs risk and protective indicators with more focus on YLWH across demographic, psychosocial and also HIV-related clinical factors. To the best of our knowledge, this is the first study in Kenya comparing the burden of CMDs between YLWH and their uninfected peers.

## Methods

### Study design and setting

This cross-sectional study was conducted at the Kenyan Coast in Kilifi and Mombasa Counties, between November 2018 and September 2019. Kilifi County is mostly a rural setting with an estimated population of 1.5 million people [31]. The main economic activity in this County is subsistence farming and fishing [32]. HIV prevalence among individuals aged 15 years and above in Kilifi County is estimated at 4.5%, slightly lower than the national average prevalence of 6% [33]. Mombasa County borders Kilifi County to the north. It is an urban County where one of the three major cities of Kenya (Mombasa) is located, and has an estimated population of 1.2 million people [31]. Because of urbanicity, the County consists of a mix of local and immigrant communities from other parts of Kenya. The main economic activity in this County is tourism, which contributes about 68% of the employment wage [34]. HIV prevalence among individuals aged 15 years and above in this County is estimated at 7.5%, 1.2 times higher than the national prevalence [33].

### Study participants and recruitment

#### Young people living with HIV

YLWH were recruited from 20 public HIV specialized clinics in Kilifi (*n* = 13) and Mombasa Counties (*n* = 7) through consecutive sampling. These clinics were selected carefully to ensure regional representativeness and a fair pool of urban/peri-urban clinics and remote/rural clinics. As inclusion criteria, individuals had to be 18–24 years old, with a confirmed HIV-positive status, on cART and provide informed consent for their participation. Pregnancy is a time of increased vulnerability for developing depression and anxiety and the risk may particularly be elevated if the pregnancy is unwanted or unplanned [35]. Therefore, we excluded females whose medical records indicated they were pregnant or those who self-reported being pregnant upon enquiry to minimize the potential for overestimating the prevalence of our outcomes of interest (depressive and anxiety symptoms).

In all facilities, we used existing records to identify all potential participants assisted by peer educators or health care providers. An effort was made to contact all those who had available working contact details (in an alphabetic order) to invite them to study briefs on a day coinciding with the structured monthly teen support group meetings. Those who could not be reached via the provided mobile contacts or had no contact details were traced during their next scheduled clinic appointment dates. Study introductions were done on one or multiple occasions (depending on facility volume). Recruitments and bookings for assessment were performed on those who consented after being taken through the study in details by research assistants present at the time of the meeting. Throughout the data collection period, recruitment was conducted and completed in one facility before moving to the next facility. We stopped recruiting at a facility when no more new, eligible and consenting study participants could be identified.

#### Young people without HIV

Young people without HIV, hereafter referred to as community controls, were recruited mainly through consecutive sampling following study advertisements on posters and flyers. Recruitment was restricted to communities adjacent to the facility from where we recruited YLWH. To be included in the study, individuals had to be 18–24 years old, residents of Kilifi or Mombasa Counties and provide consent for participation, including willingness to self-test for HIV using an oral self-testing kit (OraQuick) for a confirmation of HIV negative status. Like YLWH, we excluded females who self-reported being pregnant upon enquiry.

**Fig. 1:**
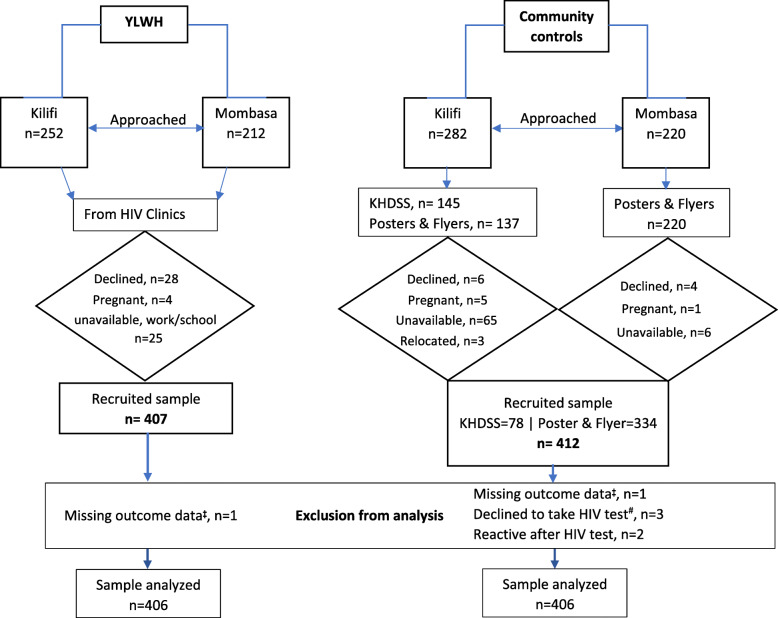
Participant recruitment flowchart. YLWH – Young People Living With HIV/AIDS. KHDSS - Kilifi Health and Demographic Surveillance System. ^**‡**^ Due to a technical error on the windows tablet data capture platform, and participants could not be reached on contact details provided for re-assessment. ^#^ HIV testing was the last step of community control assessments, and these participants declined to be tested even though they initially consented

In Kilifi County, other than the use of posters and flyers to recruit participants, we also recruited some participants through the Kilifi Health and Demographic Surveillance System (KHDSS) [32]. In Mombasa County, recruitment was solely based on the informational materials distributed in the communities as currently there is no existing surveillance system. Under KHDSS recruitment, potential participants 18–24 years old were randomly identified from the existing database and followed-up at their homesteads by our research assistants for potential recruitment. The study information sheet was given or explained to those who showed interest of participation (in English or the national language – Kiswahili, as preferred). Consenting and booking for assessments was done on site to those willing to participate. On the day of assessment, re-affirming consent was done as the first thing before any assessment began by a different research assistant. Figure 1 details the recruitment process.

### Sample size calculations

For prevalence estimates, the sample size was calculated using a previously reported significant difference in the prevalence estimate of depressive symptoms between those living with HIV and community controls [36]. A total sample of at least 718 individuals was required to detect a difference in CMDs between YLWH and community controls at 80% power and 5% level of statistical significance. A sample of > 800 participants was considered sufficient to allow for missing data, non-contact, or other factors that tend to reduce the final sample size. A one-group sample (*n* = 400) from the above sample computation was > 90% powered to carry out a logistic regression analysis of the correlates of CMDs based on previously reported effect sizes from a study conducted in a similar setting [37].

### Measures

Study instruments that do not ask about sensitive personal information were programmed on android tablets using Research Electronic Data Capture (REDCap) platform [38] for face-to-face interviewer administration. Study instruments asking about sensitive information were completed using Audio Computer-Assisted Self Interview (ACASI) on Windows tablets. In ACASI, participants privately listened to pre-recorded survey questions using headphones attached to a Windows tablet. ACASI is preferred for responding to interview questions as it improves privacy, confidentiality and reduces social desirability bias [39].

#### Interviewer-administered measures via android tablets

To administer the following tablet-based questionnaires, research assistants received a week-long training from the first author (MKN). The training was on study research methods, ethical research principles involving humans as participants, and good interviewing skills (with role plays).

*Sociodemographic and asset index forms.* The sociodemographic form captured participant’s age, sex, marital status, educational level, employment status, the living status of parents and whom they currently lived with. The asset index form gathered information about individual or family ownership of disposable items as a proxy indicator of socioeconomic status.

*The 24-item Social Provisions Scale* [40] was used to tap perceived social support. This scale is rated on a 4-point Likert scale with anchors of “1” (strongly disagree) and “4” (strongly agree). Item scores are summated to derive a total score ranging between 24 to 96; higher scores indicating better perceived social support. In the present study, the internal consistency alpha for this scale was good, 0.84 (95% CI 0.82, 0.86).

*The RAND 36-Item Self-report form* [41] was used to assess health-related quality of life. This scale assesses health concepts in the domains of: physical functioning, role limitations due to physical health or emotional problems, social functioning, emotional wellbeing, energy/fatigue, pain and general health perceptions. According to the scoring method [41], items are scored on varying Likert-type scales per domain, then summed and averaged. Higher scores (as percentages) define a more favourable state of health. We have previously used this scale in our setting with excellent reliability [29]. In the present study, the internal consistency alpha for this scale was excellent, 0.93 (95% CI 0.92, 0.94).

*HIV-related health history and clinical records.* For YLWH only, HIV-specific questions were asked related to the presence of any current chronic illness, opportunistic infection or cART side effects (as informed by their clinician), disclosure of HIV status, clinic accessibility and their satisfaction with the level of care they currently received. Data regarding World Health Organization (WHO) HIV clinical staging, recent body mass index, current cART regimen and overall duration on cART were extracted from medical records and health passports of participating YLWH and uploaded on the tablet’s electronic data capture platform. We also collected blood samples from YLWH for definitive viral load testing.

#### Measures administered via audio computer-assisted self-interview (ACASI)

Participants first received a tutorial on how to use the Windows tablet in answering questions. They had an option of choosing English or Kiswahili as their preferred language for answering the questions. An option of “*I do not want to answer the question*” was also provided in-line with respecting a client’s right of not responding to questions they do not wish to. There was also an “*assisted*” version in Kiswahili language for individuals unable (uncomfortable) to respond on their own due to e.g. illiteracy. For such cases (*n* = 19), a trained research assistant sat with the participant throughout the survey to explain, clarify or translate into local language any word, phrase or sentence as needed. In anticipation of dealing with less literate participants, research assistants were coached (with role plays) on how to help participants comprehend questions and giving them time and space to respond on their own. The assistants were also required to make their role clear before the start of an interview session. Participants were informed about our standby study counsellor for debriefing if they experienced distress as a result of completing the survey questions.

*Mental health measures.* The 9-item Patient Health Questionnaire (PHQ-9) [42] and the 7-item Generalized Anxiety Disorder scale (GAD-7) [43] were used to measure depressive and anxiety symptoms, respectively. Items in these measures are rated on a 4-point Likert scale of “0” (not at all) to “3” (nearly every day). Item scores are summated to derive a total score that ranges from 0 to 27 for PHQ-9, and 0–21 for GAD-7. For PHQ-9, total scores of 5–9, 10–14, 15–27 indicate mild, moderate, and severe depressive symptoms, respectively. GAD-7 scores of 5–9, 10–14, and 15–21 indicate mild, moderate, and severe anxiety symptoms, respectively. The recommended optimal cut-off score of ≥10 for both PHQ-9 and GAD-7 [42, 43], also applicable in sub-Saharan Africa [44, 45], was used to define positive screen for depressive and anxiety symptoms. PHQ-9 and GAD-7 have locally been validated, showing good psychometric properties [46, 47]. In the present study, the internal consistency alphas for PHQ-9 and GAD-7 were all good, 0.83 (95% CI 0.81, 0.86), and 0.86 (95% CI 0.84, 0.88), respectively.

*Negative life events score index.* A 15-item scale of negative life events was assembled adapting items from the life events questionnaire [48] and from a previous study [49]. Items covered individual-related negative events (e.g. severe illness, lack of basic needs, financial worries), negative events in the domains of school (e.g. quitting school), relationships and love (e.g. infidelity, break-ups), family, close friends and relatives (e.g. bereavement), crime and legal matters (e.g. if ever been robbed or jailed). Respondents were asked to indicate whether they experienced such negative events in the past 1 year on a dichotomous scale (yes/no). A total score was generated to reflect the total number of life events reported. In the present study, this scale had an acceptable internal consistency alpha, 0.74 (95% CI 0.71, 0.77).

*The brief 12-item HIV stigma scale* [50] was used to assess participants perceived HIV-related stigma rated on a 4-point Likert scale as “1” (strongly disagree), “2” (disagree) “3” (agree) and “4” (strongly agree). Item scores are summated to derive a total score that ranges between 12 and 48, higher scores indicating a greater level of perceived stigma. We have previously used this scale in our setting with good reliability alpha [51]. In the present study, this scale had good internal consistency alpha, 0.81 (95% CI 0.78, 0.84).

*The 4-item Morisky, Green and Levine Medication Adherence Scale (MGLS)* [52] was used as a self-report measure of cART adherence. The MGLS has a total score ranging from 0 to 4 and based on this score, there are 3 levels of medication adherence: high (a score of 0), medium (scores of 1 or 2), and low adherence (scores ≥3). In the original validation of MGLS, its reliability was acceptable and concurrent and predictive validities were established [52]. In this study, MGLS had acceptable internal consistency alpha, 0.69 (95% CI 0.62, 0.76).

### Cross-cultural adaptation of study measures

In this study, all questionnaires not previously adapted to the local language of Kiswahili (the social provisions scale, the negative life events index score and the MGLS) underwent the recommended cross-cultural validation process [53] and in line with international guidelines in health research (https://www.who.int/substance_abuse/research_tools/translation/en/). These measures were independently translated from English to Kiswahili by two staff members fluent in both languages, then back-translations into English were done by another independent pair of translators. A group of HIV researchers in the department (natives of Kenya, knowledgeable about the Kenyan culture, bilingual and fluent in English and Kiswahili) and the translators then held a harmonization meeting to review the content, conceptual and cultural appropriateness of the questions to the targeted sample. Discrepancies in the translations were resolved by consensus. No issues arose during the tool pretesting stage to necessitate item modification, and these measures were therefore regarded as the final Kiswahili versions.

### Statistical analysis

Data were analysed for 812 participants. All analyses were conducted in STATA version 15.0 (StataCorp LP, College Station, Texas, United States of America [USA]). The choice of variable reference category in the analysis of this work was informed by variable categorization in previous research on this topic.

#### Summary of data by participant group

Descriptive statistics were used to summarize sample characteristics by HIV status. Chi-square test and independent Student’s t-test were used to compare group differences on categorical and continuous independent variables, respectively.

#### Analysis of prevalence data

Proportions as percentages were used to estimate the prevalence of CMDs (depressive symptoms, anxiety symptoms, and their comorbidity) among YLWH and their uninfected peers. A positive screen for any CMD was defined using the recommended cut-off score of ≥10. Chi-square test and two-sample test of proportions (prtest) were used to compare group differences on ordinal and binary outcome variables, respectively.

#### Analysis of risk and protective indicators for CMDs

To investigate HIV status as an independent predictor of CMDs, we used logistic regression analyses adjusting for contextual variables that accounted for differences in mental health (sex, education level, employment status, parental loss and negative life events). Investigation of correlates of CMDs applied logistic regression models to assess univariate associations between the binary outcome variables (depressive symptoms, anxiety symptoms and their comorbidity) and exposure variables (demographic, psychosocial, and HIV-related clinical factors). Exposure variables having a *p*-value < 0.15 in the univariate analysis were entered into the multivariable logistic regression models using forward selection. All multivariable models included age and sex as a priori factors found to be associated with CMDs from the literature. In all the models, collinearity diagnostics were performed using STATA’s ‘collin’ syntax and based on the variance inflation factor, no multicollinearity problems were identified. For all tests of hypothesis, a two-tailed *p*-value of < 0.05 was considered statistically significant, with a confidence interval of 95% used to report on the precision of the reported estimates. The overall fit of the final models was assessed by Hosmer and Lemeshow goodness of fit test, where *p*-value > 0.05 was regarded a good fit. Data for YLWH and community controls were separately analysed.

## Results

### Overall sample characteristics

Table 1 shows results from a descriptive analysis of participant demographic and psychosocial characteristics disaggregated by HIV status. Statistical group differences are also presented. In summary, the mean age of the participants was 20.9 (SD = 2.1) years, with 50.7% being females. There was a near equal number of participants from both Counties. A majority (82.7%) of these young people were never married. Only 1.5% of them had no formal education. Most of the study participants were either students (37.3%) or unemployed (47.8%). About 18% of these young people reported that both their parents had died (mostly YLWH, *n* = 132 than community controls, *n* = 13). Over three-quarters (88.7%) of the study participants lived with immediate family or with a relative at the time of data collection. Over a quarter (31.5%) reported having experienced 6 or more negatives events in their life in the past 1 year.

**Table 1:** Sociodemographic and psychosocial characteristics of young people from the Kenyan coast by HIV status, *n* = 812

Characteristic	Total sample ***n*** = 812	HIV status		
		HIV uninfected youths, ***n*** = 406	HIV infected youths, ***n*** = 406	***p***-value#
**Study sites**				
Kilifi	403 (49.6)	200 (49.3)	203 (50.0)	0.83
Mombasa	409 (50.4)	206 (50.7)	203 (50.0)	
**Age** -years, mean (SD)	20.9 (2.1)	21.0 (1.9)	20.8 (2.2)	0.13
**Sex**				
Male	400 (49.3)	224 (55.2)	176 (43.4)	< 0.01
Female	412 (50.7)	182 (44.8)	230 (56.7)	
**Marital status**, OM = 2				
*Never married*	670 (82.7)	358 (88.6)	312 (76.9)	< 0.01
*Separated/Divorced/Widowed*	32 (4.0)	8 (2.0)	24 (5.9)	
*Married/cohabiting*	108 (13.3)	38 (9.4)	70 (17.2)	
**Education level**				
*Tertiary*	193 (23.8)	130 (32.0)	63 (15.5)	< 0.01^**†**^
*Secondary*	354 (43.6)	179 (44.1)	175 (43.1)	
*Primary*	253 (31.2)	93 (22.9)	160 (39.4)	
*None*	12 (1.5)	4 (1.0)	8 (2.0)	
**Employment**				
Formally employed	27 (3.3)	11 (2.7)	16 (3.9)	0.16
Self-employed	94 (11.6)	52 (12.8)	42 (10.3)	
Student	303 (37.3)	162 (39.9)	141 (34.7)	
Unemployed	388 (47.8)	181 (44.6)	207 (51.0)	
**Parental loss**				
*Both parents alive*	429 (52.8)	308 (75.9)	121 (29.8)	< 0.01
*One parent alive*	238 (29.3)	85 (20.9)	153 (37.7)	
*Both parents died*	145 (17.9)	13 (3.2)	132 (32.5)	
**Living arrangement**				
*Family/Relative*	720 (88.7)	359 (88.4)	361 (88.9)	0.91
*Friend/non-relative*	17 (2.1)	8 (2.0)	9 (2.2)	
*Alone*	75 (9.2)	39 (9.6)	36 (8.9)	
**Negative life events**				
*None*	80 (9.9)	51 (12.6)	29 (7.1)	< 0.01
*1–5 events*	476 (58.6)	255 (62.8)	221 (54.4)	
*6+ events*	256 (31.5)	100 (24.6)	156 (38.4)	
**Asset index score** ^a^ – mean (SD)	2.4 (1.6)	2.6 (1.6)	2.2 (1.6)	< 0.01
**Social support score** ^b^ – mean (SD)	71.6 (8.7)	72.1 (7.8)	71.1 (9.5)	0.09
**Quality of life score** ^c^ – mean (SD)	78.8 (15.3)	82.7 (12.0)	74.8 (17.2)	< 0.01

All numbers are reported as frequencies with percentages unless otherwise specified

# *p*-values are for the difference between HIV infected and uninfected youths by sample characteristic

^**†**^ based on Fisher’s exact test, *SD* standard deviation, *OM* observation with missing value, ^a^ – score range = 0 to 7, higher scores indicate better socioeconomic status, ^b^ – score range = 24 to 96, higher scores indicate more perceived social support, ^c^ – higher scores indicate better state of perceived health

Significant sociodemographic differences were observed between YLWH and their uninfected peers in terms of sex, marital status and education level, all *p* < 0.01 (Table 1).

### HIV-related clinical and psychosocial characteristics of YLWH

Table 2 shows results from a descriptive analysis of HIV-related clinical and psychosocial characteristics of YLWH. In summary, their mean body mass index was within the normal range (mean [SD] = 20.6 [3.6]). Most of these YLWH were on cART for over 5 years (57.9%), largely first-line regimen (81.3%) and were highly adherent based on self-report (62.6%). Over half were in stage 1 of WHO clinical staging of HIV (61.5%) and had viral load *≤*1000 copies/mL (69.0%). More than three-quarters of YLWH were generally satisfied with the current level of care they were receiving (94.3%), had disclosed their HIV status (93.8%), reported no current comorbid chronic illness (98.3%), reported absence of any current opportunistic infection (93.8%) or medication-related side-effects (73.1%). A quarter of YLWH (25.1%) reported their present HIV point of care as not easily accessible.

**Table 2:** HIV-related clinical and psychosocial characteristics of YLWH from the Kenyan coast, *n* = 406

Characteristic	Mean/Frequency	SD/percent
**Recent Body Mass Index** – kg/m^2^, mean, SD	20.6	3.6
**Any current chronic illness**		
*No*	399	98.3
*Yes* ^*†*^	7	1.7
**cART regimen**		
*First line*	330	81.3
*Second line* ^*1*^	76	18.7
**Viral load**		
*≤ 1000 copies/mL*	280	69.0
*> 1000 copies/mL*	126	31.0
**WHO clinical stage**, OM = 1		
*Stage 1*	249	61.5
*Stage 2*	102	25.2
*Stage 3*	52	12.8
*Stage 4*	2	0.5
**Duration on cART**		
*> 5 years*	235	57.9
*1–5 years*	124	30.5
*6-11 months*	25	6.2
*< 6 months*	22	5.4
**Any current opportunistic infection**		
*No*	381	93.8
*Yes*	25	6.2
**Any cART side effects**, OM = 1		
*No*	296	73.1
*Yes*	109	26.9
**Perceived HIV-stigma score** ^a^ – mean, SD	26.1	7.5
**Medication (cART) adherence**		
*High adherence*	254	62.6
*Medium adherence*	117	28.8
*Low adherence*	35	8.6
**HIV status disclosure**		
*Yes*	381	93.8
*No*	25	6.2
**Clinic accessibility**		
*Easily accessible (Less than 30 min)*	129	31.8
*Somehow accessible (30 min to 1 h)*	175	43.1
*Not accessible (> 1 h)*	102	25.1
**Satisfaction with current care**		
*Satisfied*	383	94.3
*Neutral*	16	3.9
*Not satisfied*	7	1.7

^a^ – score range = 12 to 48, higher scores indicate increased perceived HIV-related stigma, ^†^ one or more chronic illness in addition to HIV, *OM* observation with missing value, *WHO* world health organization, *cART* combination antiretroviral therapy, ^1^- all participants were initially started on 1st line cART but had changed to 2nd line cART at the time of data collection

### Prevalence of common mental disorders

Table 3 presents prevalence estimates for CMDs among YLWH compared to their HIV-uninfected peers. The prevalence of CMDs was significantly higher among YLWH compared to community controls in terms of severity levels and positive screening using the recommended cut-off score of ≥10. The prevalence of a positive screen for depressive symptoms was 28.8% among YLWH compared to 12.1% in community controls (*p* < 0.001). The prevalence of a positive screen for anxiety symptoms was 19.0% among YLWH compared to 7.6% in community controls (*p* < 0.001). The prevalence of comorbid positive screen for depressive and anxiety symptoms among YLWH compared to community controls was 16.0% vs. 4.7% (*p* < 0.001).

**Table 3:** Prevalence of common mental disorders in YLWH versus their uninfected peers from the Kenyan coast

	HIV uninfected youths, ***n*** = 406		HIV infected youths, ***n*** = 406		***p***-value
	Frequency	Prevalence (95% CI)	Frequency	Prevalence (95% CI)	
**Severity of depressive symptoms**					
Mild	86	21.2 (17.5, 25.4)	130	32.0 (27.6, 36.7)	< 0.001^†^
Moderate	34	8.4 (6.0, 11.5)	78	19.2 (15.7, 23.4)	
Moderately severe	12	3.0 (1.7, 5.1)	30	7.4 (5.2, 10.4)	
Severe	3	0.7 (0.2, 2.3)	9	2.2 (1.2, 4.2)	
**Positive depression screen (cut-off score ≥ 10)**					
Yes	49	12.1 (9.2, 15.6)	117	28.8 (24.6, 33.4)	< 0.001^#^
**Severity of GAD symptoms**					
Mild	98	24.1 (20.2, 28.6)	138	34.0 (29.5, 38.8)	< 0.001
Moderate	20	4.9 (3.2, 7.5)	54	13.3 (10.3, 17.0)	
Severe	11	2.7 (1.5, 4.8)	23	5.7 (3.8, 8.4)	
**Positive GAD screen (cut-off score ≥ 10)**					
Yes	31	7.6 (5.4, 10.7)	77	19.0 (15.4, 23.1)	< 0.001^#^
**Positive screen for comorbid depressive and GAD symptoms**					
Yes	19	4.7 (3.0, 7.2)	65	16.0 (12.7, 19.9)	< 0.001^#^

*95% CI* 95% confidence interval, *GAD* Generalized anxiety disorder

^†^ based on Fisher’s exact test

^#^ based on prtest, a two-sample test of differences in proportion using defined binary groups

### Association between HIV status and common mental disorders

Table 4 presents results from logistic regression analyses examining the association between HIV status (exposure) and CMDs (outcome). In univariate logistic regression analysis, being HIV positive was significantly associated with higher odds of (a positive screen for) depressive symptoms, anxiety symptoms and their co-occurrence (Table 4). Adjusting for sex, education level, employment status, parental loss and negative life events in the multivariable analysis (Table 4), being HIV positive remained significantly associated with higher odds of depressive symptoms (*aOR 1.77 95% CI 1.13, 2.79*) and co-occurrence of depressive and anxiety symptoms (*aOR 3.88 95% CI 2.28, 6.61*), but not anxiety symptoms alone (*aOR 1.51 95% CI 0.88, 2.58*).

**Table 4:** Association between HIV status and common mental disorders among young people from Kenyan coast

	Positive screen for depressivesymptoms		Positive screen for GAD symptoms		Positive CMD comorbidity ^**a**^	
Covariate	Crude analysis OR (95% CI)	Adjustedanalysis^**b**^ aOR (95% CI)	Crude analysis OR (95% CI)	Adjustedanalysis^**b**^ aOR (95% CI)	Crude analysis OR (95% CI)	Adjustedanalysis^**b**^ aOR (95% CI)
**HIV status**						
Negative	Ref	Ref	Ref	Ref	Ref	Ref
Positive	2.95** (2.04, 4.26)	1.77* (1.13, 2.79)	2.83** (1.82, 4.41)	1.51 (0.88, 2.58	3.88** (2.28, 6.61)	2.07* (1.11, 3.88)
**Sex**						
Male		Ref		Ref		Ref
Female		1.41 (0.95, 2.09)		1.70* (1.06, 2.72)		2.16* (1.26, 3.70)
**Education**						
*Secondary*		Ref		Ref		Ref
*Tertiary*		1.16 (0.68, 1.97)		1.56 (0.84, 2.91)		1.77 (0.88, 3.56)
*Primary*		1.77* (1.13, 2.76)		1.67 (0.98, 2.83)		2.12* (1.17, 3.84)
*None*		2.20 (0.52, 9.27)		2.33 (0.49, 11.04)		0.78 (0.08, 7.31)
**Employment**						
Student		Ref		Ref		Ref
Self-employed		0.68 (0.33, 1.38)		1.24 (0.55, 2.79)		0.62 (0.23, 1.67)
Formally employed		1.10 (0.40, 3.00)		1.95 (0.65, 5.80)		1.59 (0.50, 5.13)
Unemployed		1.14 (0.73, 1.80)		1.45 (0.83, 2.54)		1.12 (0.61, 2.06)
**Parental loss**						
Both parents alive		Ref		Ref		Ref
One parent alive		1.32 (0.83, 2.09)		1.83* (1.06, 3.15)		1.83 (0.99, 3.39)
Both parents died		1.80* (1.06, 3.06)		2.40* (1.30, 4.44)		2.26* (1.15, 4.45)
**Negative life events**						
None^**c**^		0.20* (0.05, 0.87)		1.00		1.00
1–5 events		Ref		Ref		Ref
6+ events		4.26** (2.89, 6.27)		4.00** (2.54, 6.31)		3.80** (2.28, 6.35)
**n of the final model**		812		732		732
**Hosmer-LemeshowTest**		X^2^ = 218.73; *p* = 0.26		X^2^ = 168.42; *p* = 0.48		X^2^ = 170.68; *p* = 0.43
**Variance explained**		17.1%		15.0%		16.7%

*GAD* Generalized anxiety disorder, *CMD* Common mental disorder, *OR* odds ratio, *aOR* adjusted odds ratio, *Ref* reference group

* - *p* value< 0.05, ** - *p* value <0.001

^a^ - co-occurrence of positive screen for depressive and anxiety symptoms

^b^ − adjusted for sex, level of education, employment, death of parent(s) and negative life events (in the past 1 year)

^c^ − for this category, no participant screened positive for anxiety or comorbidity symptoms, hence predicted failure perfectly and consequently 80 observations were not used

Repeating this analysis by including all variables associated with CMDs at *p* < 0.15 in the multivariable logistic analysis as covariates, age and sex fixed (data not shown), findings were similar. Being HIV positive was significantly associated with higher odds of depressive symptoms, its co-occurrence with anxiety symptoms, but not anxiety symptoms alone. Additionally, experiencing 6 or more negative life events was significantly associated with higher odds of depressive symptoms (*aOR 2.82 95% CI 1.87, 4.26*), anxiety symptoms (*aOR 2.67 95% CI 1.64, 4.34*), and their co-occurrence (*aOR 2.35 95% CI 1.36, 4.06*). Death of both parents was significantly associated with nearly 2-times higher odds of anxiety symptoms (*aOR 1.91 95% CI 1.05, 3.49*). Being female was also significantly associated with higher odds of co-occurrence of depressive and anxiety symptoms (*aOR 1.89 95% CI 1.08, 3.33*). Higher social support and health-related quality of life were significantly associated with lower odds of a positive screen for CMDs.

### Risk and protective indicators of common mental disorders in YLWH

Table 5 presents results from logistic regression analyses examining the correlates (both risk and protective indicators) of CMDs among YLWH. Under univariate analysis, factors that were significantly associated with higher odds of (a positive screen for) depressive symptoms among YLWH included: increasing age, being female, having primary education (relative to secondary education), being unemployed, death of both parents, experiencing 6 or more negative life events (in the past 1 year), low and medium adherence to cART, perceived HIV-related stigma and presence of cART side effects (*p* < 0.05; Table 5). Higher social support and health-related quality of life were significantly associated with lower odds of depressive symptoms. High viral load (> 1000 copies/mL) and being on cART for less than 6 months were associated with a positive screen for depressive symptoms at *p*-value < 0.15 and were all included in multivariable logistic regression analysis. In the multivariable model, with age and sex fixed, factors significantly associated with higher odds of depressive symptoms among YLWH were negative life events (6 or more) and perceived HIV-related stigma (Table 5). Higher social support and health-related quality of life were significantly associated with lower odds of depressive symptoms (Table 5).

**Table 5:** Univariate and multivariable analysis of correlates of common mental disorders among YLWH from Kenyan coast

	Positive screen for depressive symptoms		Positive screen for GAD symptoms		Positive CMD Comorbidity^**a**^	
Covariate	Univariate analysis OR (95% CI)	Multivariable analysis aOR (95% CI)	Univariate analysis OR (95% CI)	Multivariable analysis aOR (95% CI)	Univariate analysis OR (95% CI)	Multivariable analysis aOR (95% CI)
**A priori:**						
**Age**	1.12** (1.02, 1.24)	1.02 (0.91, 1.15)	1.09* (0.97, 1.22)	1.01 (0.88, 1.16)	1.14** (1.01, 1.29)	1.06 (0.92, 1.23)
**Sex**						
Male	Ref	Ref	Ref	Ref	Ref	Ref
Female	1.62** (1.04, 2.53)	1.08 (0.63, 1.84)	2.04*** (1.20, 3.48)	1.41 (0.74, 2.66)	2.07** (1.16, 3.67)	1.45 (0.73, 2.85)
**Demographic:**						
**Education level**						
*Secondary*	Ref	–	Ref	–	Ref	–
*Tertiary*	1.25 (0.65, 2.41)	–	1.41 (0.66, 3.01)	–	1.74* (0.78, 3.89)	–
*Primary*	1.87** (1.16, 3.01)	–	1.87** (1.07, 3.27)	–	2.22** (1.21, 4.07)	–
*None*	2.03 (0.46, 8.84)	–	2.00 (0.38, 10.47)	–	1.17 (0.14, 10.06)	–
**Employment**						
Student	Ref	–	Ref	–	Ref	–
Self-employed	0.71 (0.29, 1.75)	–	0.99 (0.34, 2.85)	–	0.77 (0.24, 2.42)	–
Formally employed	1.61 (0.52, 4.99)	–	3.32* (1.03, 10.71)	–	2.43 (0.70, 8.40)	–
Unemployed	1.97*** (1.21, 3.22)	–	2.32*** (1.28, 4.23)	–	1.75* (0.95, 3.23)	–
**Living arrangement**						
Family/Relative	–	–	Ref	–	Ref	–
Friend/non-relative	–	–	0.58 (0.07, 4.72)	–	0.73 (0.09, 5.93)	–
Alone	–	–	2.32** (1.10, 4.88)	–	2.56** (1.19, 5.50)	–
**Psychosocial:**						
**Parental loss**		–		–		–
Both parents alive	Ref	–	Ref	–	Ref	–
One parent alive	1.41 (0.81, 2.44)	–	2.03* (1.01, 4.08)	–	1.86* (0.90, 3.86)	–
Both parents died	1.86** (1.07, 3.25)	–	2.88*** (1.44, 5.78)	–	2.34** (1.12, 4.85)	–
**Negative life events**						
None^b^	0.16* (0.02, 1.22)	0.26 (0.03, 2.02)	1.00	–	1.00	–
1–5 events	Ref	Ref	Ref	–	Ref	–
6+ events	4.30*** (2.70, 6.84)	2.53*** (1.50, 4.26)	3.64*** (2.15, 6.18)	–	3.44*** (1.96, 6.05)	–
**Asset index**	–	–	0.81** (0.68, 0.95)	–	0.76*** (0.63, 0.92)	–
**Social support**	0.94*** (0.92, 0.96)	0.96*** (0.93, 0.98)	0.93*** (0.91, 0.96)	0.95*** (0.92, 0.98)	0.92*** (0.89, 0.95)	0.94*** (0.91, 0.97)
**Health-related quality of life**	0.95*** (0.93, 0.96)	0.96*** (0.95, 0.98)	0.94*** (0.93, 0.96)	0.96*** (0.94, 0.97)	0.95*** (0.93, 0.96)	0.96*** (0.94, 0.98)
**Medication adherence**						
High adherence	Ref	–	Ref	Ref	Ref	Ref
Medium adherence	2.18*** (1.35, 3.52)	–	2.01** (1.14, 3.53)	1.58 (0.82, 3.04)	2.08** (1.15, 3.78)	1.76 (0.88, 3.52)
Low adherence	3.83*** (1.85, 7.93)	–	6.32*** (2.97, 13.49)	2.97** (1.27, 6.95)	4.77*** (2.16, 10.51)	2.49** (1.02, 6.06)
**Perceived HIV-stigma**	1.10*** (1.06, 1.13)	1.04** (1.01, 1.08)	1.13*** (1.09, 1.17)	1.09*** (1.04, 1.14)	1.12*** (1.07, 1.16)	1.07*** (1.02, 1.12)
**Clinical:**						
**Body Mass Index**	–	–	1.05* (0.99, 1.12)	–	–	–
**Viral load**						
≤ 1000 copies/mL	Ref	–	–	–	–	–
> 1000 copies/mL	1.44* (0.92, 2.27)	–	–	–	–	–
**Duration on cART**						
> 5 years	Ref	–	–	–	–	–
1–5 years	1.07 (0.66, 1.75)	–	–	–	–	–
6-11 months	1.54 (0.65, 3.65)	–	–	–	–	–
< 6 months	2.28* (0.94, 5.53)	–	–	–	–	–
**cART side effects**						
No	Ref	–	–	–	–	–
Yes	1.75** (1.09, 2.78)	–	–	–	–	–
**n for the final model**		406		406		406
**Variance explained**		22.3%		26.2%		25.0%
**Hosmer-Lemeshow test**		X^2^ = 380.22; *p*-value = 0.73		X^2^ = 437.56; *p*-value = 0.08		X2 = 402.55; *p*-value = 0.43
**cvMean AUC (95% CI)**		0.78 (0.69, 0.81)		0.81 (0.77, 0.88)		0.81 (0.74, 0.86)

Only a priori variables (age, sex), as well as those with *p*-value < 0.15 in the univariate analysis or multivariable *p* < 0.05 are presented here

*GAD* Generalized anxiety disorder, *CMD* common mental disorder, *OR* odds ratio, *aOR* adjusted odds ratio, *Ref* reference group, *cvMean AUC* cross-validated mean area under the curve for the final multivariable model

* - *p* value< 0.15, ** - *p* value < 0.05, *** - *p* value < 0.01

^**a**^ - co-occurrence of positive screen for depressive and anxiety symptoms

^b^ − for this category, no participant screened positive for anxiety or comorbidity symptoms, hence in the univariate analysis it predicted failure perfectly

Factors that were significantly associated with higher odds of (a positive screen for) anxiety symptoms among YLWH in the univariate analysis included: being female, having primary education, being unemployed, living alone, death of both parents, experiencing 6 or more negative life events, low and medium adherence to cART, and perceived HIV-related stigma (*p* < 0.05; Table 5). Having more assets (a proxy for better socioeconomic status), higher social support and health-related quality of life were significantly associated with lower odds of anxiety symptoms. Increasing age and body mass index were associated with a positive screen for anxiety symptoms at *p*-value < 0.15 and were all included in multivariable analysis. Fixing age and sex as constant in the multivariable models (Table 5), low adherence to cART and perceived HIV-related stigma were significantly associated with higher odds of anxiety symptoms among YLWH. Higher social support and health-related quality of life remained significantly associated with lower odds of anxiety symptoms (Table 5).

Factors that were significantly associated with higher odds of (a positive screen for) comorbid depressive and anxiety symptoms among YLWH in the univariate analysis included: increasing age, being female, having primary education, living alone, death of both parents, experiencing 6 or more negative life events, low and medium adherence to cART, and perceived HIV-related stigma (*p* < 0.05; Table 5). Having more assets, higher social support and health-related quality of life were significantly associated with lower odds of comorbid depressive and anxiety symptoms. Fixing age and sex as constant in the multivariable logistic models (Table 5), low adherence to cART and perceived HIV-related stigma were significantly associated with higher odds of comorbid depressive and anxiety symptoms among YLWH. Higher social support and health-related quality of life remained significantly associated with lower odds of comorbid depressive and anxiety symptoms (Table 5).

### Risk and protective indicators of common mental disorders in HIV-uninfected community controls: a snapshot summary

This work mostly focused on YLWH. As such, data collected from HIV-uninfected young people was not as robust as that collected from YLWH to comprehensively look at factors associated with CMDs. For instance, data about community experiences or health aspects of HIV-uninfected peers were not collected. Here, we only provide a snapshot summary of the correlates of CMDs among community controls using the available data specifically focusing on depressive symptoms whose prevalence was relatively high in this sample (> 10%).

Under multivariable analysis (detailed data not shown), the death of both parents (*aOR 4.42 95% CI 1.15, 17.02*) was significantly associated with higher odds of (a positive screen for) depressive symptoms among community controls. Higher social support (*aOR 0.93 95% CI 0.88, 0.98*) and health-related quality of life (*aOR 0.92 95% CI 0.90, 0.95*) were significantly associated with lower odds of depressive symptoms.

## Discussion

This study is among the few emerging reports from sub-Saharan Africa investigating CMDs, specifically depressive and anxiety symptoms, in YLWH compared to their HIV-uninfected peers. In summary, we report significantly higher prevalence of CMDs in YLWH compared to their uninfected peers. We found HIV status an independent predictor of depressive symptoms and its comorbidity with anxiety symptoms. Six or more negative life events (in the past 1 year) and perceived HIV-related stigma were significant risk indicators for elevated depressive symptoms in YLWH. Low medication adherence and perceived HIV-related stigma were significant risk indicators for elevated anxiety symptoms and its comorbidity with depressive symptoms in YLWH. Higher social support and health-related quality of life were consistently associated with lower depressive symptoms, anxiety symptoms and their comorbidity in YLWH. The death of both parents was a significant risk indicator for the relatively high depressive symptoms observed among community controls. Like in YLWH, higher social support and health-related quality of life were significantly associated with lower depressive symptoms among community controls.

Our finding that CMDs are significantly higher in YLWH than their uninfected peers is similar to findings from a study conducted in Tanzania [28]. Among YLWH, our prevalence estimate of CMDs compares to that reported in Rwanda [54] and Tanzania [28]. It is higher than previously reported estimates from Malawi [25] and South Africa [18], but lower than estimates reported from Zimbabwe [55] and USA [5] . The differences in prevalence estimates could be due to the differences in the study setting and measures used. Compared to the prevalence of depressive symptoms among the general adult population living with HIV from our setting [51], we report a higher estimate among YLWH.

The relatively high proportion of depressive symptoms (12%) among HIV-uninfected peers from our setting cannot be ignored, especially since previous research from Kenya also documents a high proportion [56]. Although this work mostly focused on YLWH, from the analysed data, the death of both parents was a significant risk indicator for higher depressive symptoms among community controls. This finding has been previously observed elsewhere among youths [57]. Higher social support and health-related quality of life among HIV-uninfected young people were the protective indicators against elevated depressive symptoms. These factors have also been reported as protective of mental health in past reviews [58, 59].

In this study, HIV status was found to be an independent predictor of depressive symptoms and its co-occurrence with anxiety symptoms. This finding is consistent with previous research findings from sub-Saharan Africa [28] but contrasts findings from a study conducted in England by Prevost et al. [21] where HIV status was not associated with depressive or anxiety scores. The different study setting and studied population could explain differences in findings. In particular, the control participants in Prevost et al. [21] study were HIV-exposed uninfected youths. These youths may be experiencing similar circumstances as YLWH by virtue of coming from an HIV affected family, hence the lack of significant differences in mental health manifestation.

The odd of depressive symptoms among YLWH was 2.5 times higher as the number of negative life events in the last 1 year increased above 5. This finding is consistent with what has previously been reported in the literature [49, 60]. However, the study design we used precludes any conclusions on the dose-response relationship for this association. A cohort study among people living with HIV found no significant difference in depressive disorder among those with and without an experience of negative events [61]. Nevertheless, to help reduce the risk of depression among YLWH, those reporting considerable recent negative events in life in this setting should be targeted for early detection and prompt intervention of any CMD.

Young people 15–24 years old are at the highest risk of cART attrition compared to younger children and adults [62]. In this study, close to 9% of YLWH self-reported low adherence to cART, which was significantly associated with higher odds of anxiety symptoms and its comorbidity with depressive symptoms. Non-adherence to HIV treatment may lead to virological non-suppression due to issues such as drug resistance. Psychological distress can emerge when patients are informed about their poor prognosis. This finding is in line with previous findings [20, 63] and provides further evidence of the critical role of addressing medication adherence in improving the mental health needs of YLWH.

Perceived HIV-related stigma was significantly associated with higher odds of depressive symptoms, anxiety symptoms and their co-occurrence in this study. Other studies also report similar findings [14, 20, 23]. For a young person, stigma due to living with HIV may decrease their perceived level of social support in the community, which in turn, may increase mental ill-health symptoms. Therefore, YLWH experiencing HIV-related stigma in this or similar settings should be prioritized for mental health support.

We found that higher social support was a protective indicator against CMDs in YLWH, a finding that is supported by results from other studies [17, 22]. Social support may relieve psychological distress by improving self-esteem and decreasing negative cognition [64, 65]. In the context of living with HIV, higher social support may give an impression that one is valued and accepted by others in the society, hence reassurance of self-worth. Higher health-related quality of life was a protective indicator against CMDs in YLWH. Similarly, Boyes et al. [22] report that overall perceived better health was protective against both depressive and anxiety symptoms. In the inverse direction, other studies involving people living with HIV report significant associations between lower quality of life and elevated mental health problems [29, 66].

Since both higher social support and health-related quality of life were also protective indicators against depressive symptoms among HIV-uninfected youths in this study, community-level programmes seeking to strengthen the social capital or improve the overall quality of life of these young adults, regardless of HIV status, have the potential of improving their mental wellbeing.

In this study, sociodemographic and HIV-related clinical factors were not significantly associated with any CMD among YLWH. Similar observations have been reported within sub-Saharan Africa [19] and elsewhere [67]. In contrast, other studies from sub-Saharan Africa have found significant associations between CMDs in YLWH and demographic or HIV-related clinical factors. Kim et al. [25] found that primary education or lower was significantly associated with a 3-fold increase in depressive symptoms compared to high school education level or higher. Boyes et al. [22] report cART side effects as a risk factor for both depressive and anxiety symptoms. The presence of opportunistic infections was significantly associated with a near 2-fold increase in depressive symptoms according to a study by Abebe et al. [20]. Due to resource constraints in settings such as Kenya, more research on correlates of CMDs among YLWH is called for to identify important context-specific factors for targeted interventions at the Kenyan coast.

The strengths of the current study include a focus on an understudied but rapidly expanding population of YLWH, use of a comparison group, adequate sample size, and novel data collection methods. However, there are limitations too. The cross-sectional study design limits any causal inference for the reported significant associations. Our findings may not be generalizable to younger youths < 18 years. Relatedly, we recruited YLWH receiving care in public HIV clinics and using consecutive sampling strategy. These aspects limit the generalizability of our findings, especially to YLWH who are out of care or receiving HIV services in private facilities. We used self-report screening tools, which may be subject to reporting bias. Relatedly, the mental health screening tools do not give a clinical diagnosis of depression or anxiety. We therefore only report symptomatology of these disorders. Future studies involving YLWH should also investigate whether the prevalence of CMDs differ by mode of infection (perinatal versus behavioural) as this was beyond the scope of the present study.

### Implications of the study findings for future research, clinical care and policy

The study limitations notwithstanding, this work has important implications for future research, policy and care of young people in early adulthood. We observed a high prevalence of CMDs particularly among YLWH from the Kenyan coast and HIV status was predictive of depressive symptoms and its co-occurrence with anxiety symptoms. There is an urgent need for testing of interventions seeking to address CMDs comorbid with HIV among youths in this setting. Such interventions, whether developed or adapted, should be more youth-friendly to increase acceptability.

The high prevalence of CMDs among YLWH at the Kenyan coast also calls for routine screening of these mental disorders at the HIV clinics serving these youths. Routine screening will serve to identify affected young people early enough and initiate them on an appropriate support mechanism, including treatment or referral for specialized care. In so doing, the consequences of CMDs comorbid with HIV in young people like non-adherence to cART [68], problems with retention in care [69] and risky sexual behaviour [70], can be averted.

Compared to anxiety symptoms, the prevalence of depressive symptoms was relatively high among community controls in this study (12%). Such high prevalence could be driven by factors beyond the data that we collected from community controls and analysed. To appropriately inform policy planners, we recommend a separate study comprehensively looking at the drivers of CMDs in the general population of young adults without HIV.

Perceived HIV-related stigma and low adherence to cART significantly increased the risk for CMDs among YLWH. HIV destigmatization initiatives including multi-channel public awareness about the latest advances in the HIV/AIDS field, and cART adherence support programmes should continue taking centre-stage in this coastal setting of Kenya, as priority strategies for reducing psychiatric manifestations among these youths.

## Conclusion

At the Kenyan Coast, CMDs are more prevalent among YLWH compared to their uninfected peers. Being HIV positive as a young person in this setting is predictive of more depressive symptoms and its comorbidity with anxiety symptoms. Since early detection and management of mental health problems is key to better health outcomes, for YLWH in this setting, routine screening of these CMDs should be integrated in the care package provided to them at their point of care. Screening should prioritize YLWH at high risk of CMDs such as those who have experienced substantial negative life events or HIV-related stigma in the community, and those with antiretroviral adherence problems. Continued support to bereaving HIV-unaffected young adults could help them come to terms with their loss hence better mental wellbeing. At the community level, programmes strengthening the social capital or improving the overall quality of life of young adults with or without HIV may be beneficial to their mental health.

## Data Availability

The dataset and associated files used for analysis of this study are available in Harvard dataverse at 10.7910/DVN/4THNG4. Application for access can be made through the data governance committee of the KEMRI Wellcome Trust Research Programme who will review the application and advise as appropriate ensuring that uses are compatible with the consent obtained from participants for data collection. Requests can be sent to the coordinator of the Data Governance Committee using the following email: dgc@kemri-wellcome.org

## Abbreviations

ACASI: Audio computer assisted self-interview
cART: Combined antiretroviral therapy
CMDs: Common mental disorder
GAD-7: 7 item generalized anxiety disorder scale
KHDSS: Kilifi health and demographic surveillance system
MGLS: Morisky, Green and Levine Medication Adherence Scale
PHQ-9: 9 item patient health questionnaire
REDCap: Research electronic data capture
USA: United States of America
WHO: World Health Organization
YLWH: Young People Living with HIV
